# Complete Genome Sequence of Mesorhizobium ciceri Strain R30, a Rhizobium Used as a Commercial Inoculant for Chickpea in Argentina

**DOI:** 10.1128/mra.00779-22

**Published:** 2022-10-26

**Authors:** Emiliano Foresto, Santiago Revale, Emiliano Primo, Fiorela Nievas, Evangelina Carezzano, Mariana Puente, Pedro Alzari, Mariano Martínez, Mathilde Ben-Assaya, Damien Mornico, Maricel Santoro, Francisco Martínez-Abarca, Walter Giordano, Pablo Bogino

**Affiliations:** a Instituto de Biotecnología Ambiental y Salud (INBIAS-CONICET), Departamento de Biología Molecular, Facultad de Ciencias Exactas, Físico-Químicas y Naturales, Universidad Nacional de Río Cuarto, Río Cuarto, Córdoba, Argentina; b Wellcome Centre for Human Genetics, University of Oxford, Oxford, United Kingdom; c Instituto de Microbiología y Zoología Agrícola (IMYZA-INTA), Castelar, Buenos Aires, Argentina; d Unité de Microbiologie Structurale, Institut Pasteur, CNRS UMR 3528, Université de Paris, Paris, France; e Hub de Bioinformatique et Biostatistique, Département Biologie Computationnelle, CNRS USR 3756, Institut Pasteur, Paris, France; f Department of Biochemistry, Max Planck for Chemical Ecology, Jena, Germany; g Grupo de Ecología Genética de la Rizósfera, Estación Experimental del Zaidín (CSIC), Granada, Spain; University of Maryland School of Medicine

## Abstract

We report the complete genome sequence of Mesorhizobium ciceri strain R30, a rhizobium strain recommended and used as a commercial inoculant for chickpea in Argentina. The genome consists of almost 7 Mb, distributed into two circular replicons: a chromosome of 6.49 Mb and a plasmid of 0.46 Mb.

## ANNOUNCEMENT

As part of sustainable agriculture, legumes may be inoculated with rhizobia that develop nitrogen-fixing symbiosis with the crop and thus prevent nitrogen deficiency (1, 2). Chickpea (Cicer arietinum L.) is one of several legumes that establish symbiosis with rhizobia from the genus *Mesorhizobium* (3), more specifically with Mesorhizobium ciceri. In Argentina, the Instituto Nacional de Tecnología Agropecuaria (INTA) recommends inoculating chickpea with *M. ciceri* R30 (originally named Rhizobium ciceri USDA 3383, it was purchased in the late 1990s from USDA’s Agricultural Research Service in Beltsville, MD, USA) (4).

To date, the complete genome sequences of three *M. ciceri* strains are available at NCBI, out of seven assemblies. Only strain CC1192 is symbiotically active with chickpea (5). Here, we announce the complete annotated genome sequence of *M. ciceri* R30.

A pure culture of the strain, provided by INTA, was aerobically grown in liquid yeast extract-mannitol medium at 30°C with 150 rpm rotation until it reached the late exponential growth phase (6). This was the source for the total DNA, obtained using a DNeasy blood and tissue kit (Qiagen) for Illumina sequencing and a Wizard high-molecular-weight (HMW) DNA extraction kit (Promega) for Oxford Nanopore Technologies sequencing. The genome was assembled through a hybrid approach including short Illumina reads and long Oxford Nanopore reads. Illumina sequencing was performed on the P2M platform at Institut Pasteur. The library was prepared using a Nextera XT DNA library preparation kit and sequenced on an Illumina NextSeq 500 instrument, with a paired-end (PE) 150-bp read configuration. Nanopore sequencing was performed at the Oxford Genomics Centre. The sample was processed using an Oxford Nanopore Technologies rapid barcoding sequencing kit (SQK-RBK004) and a native barcoding genomic DNA sequencing kit (SQK-LSK109 with EXP-NBD104). The products of each were sequenced in two Flongle flow cells. Data were base called using Guppy v4.2.2, with the high-accuracy model and the –trim_barcodes option. We obtained 6,189,520 Illumina PE reads predicting 132-fold coverage and 88,970 Nanopore long reads with an *N*_50_ value of 10,948 bp and an average of 5,324 bp, predicting 68-fold coverage. Hybrid genome assembly was performed on the raw reads using the nf-core/bacass pipeline (commit ceebac0) with default parameters (7). This resulted in two contigs that were closed by manually analyzing the overlapped ends using Geneious v2019 2.1 software (8). The reported genome consists of one chromosome (6,489,234 bp; G+C content, 62.7%) and a secondary replicon from the repABC plasmid family (457,705 bp; G+C content, 59.9%) (9). The first nucleotide was assigned at the beginning of the *dnaA* and *repA* genes for the chromosome and plasmid respectively, using the corresponding tool within Geneious. Average nucleotide identity analysis (10) revealed that the R30 chromosome is >98% identical to sequenced Mesorhizobium ciceri genomes available at NCBI, such as those for *M. ciceri* strain CC1192 (GenBank accession number CP015062.1) and *M. ciceri* bv. biserrulae strains WSM1271 and WSM1284 (CP002447.1 and CP015064.1, respectively), which is evidence of their close phylogenetic relationship.

The complete genome sequence, which was annotated using the NCBI Prokaryotic Genome Annotation Pipeline (PGAP) (11–13), consists of 6,452 protein-coding sequences, 2 complete ribosomal operons, and 52 tRNAs. As in other mesorhizobia (14, 15), the genes for nodulation (*nod*) and nitrogen fixation (*nif* and *fix*) in R30 appear to be located on a chromosomic 401-kb symbiosis island (1,263,739 to 1,664,915 bp), flanked by direct repeat sequences identical to those in the ICE region in *M. ciceri* CC1192 and adjacent to one of four serine tRNA genes. This region also harbors biotin and nicotinate biosynthetic clusters, a conjugative type IV secretion system, and *luxI*- and *luxR*-like quorum-sensing genes probably associated with the island’s excision and transfer (16).

This complete sequencing of the R30 genome could be crucial for more in-depth research into its symbiotic performance and other biological features.

### Data availability.

The complete genome sequence of Mesorhizobium ciceri R30 is available at NCBI GenBank under accession numbers CP088147 for the chromosome and CP088148 for the plasmid, with BioProject accession number PRJNA782313 and BioSample accession number SAMN23371988. The raw reads are available at NCBI’s Sequence Read Archive under accession numbers SRR16992657, SRR16992658, SRR16992659, SRR16992660, and SRR16992661.
